# Recycling of Contaminated Marine Sediment and Industrial By-Products through Combined Stabilization/Solidification and Granulation Treatment

**DOI:** 10.3390/ma16062399

**Published:** 2023-03-17

**Authors:** Francesco Todaro, Francesco Colangelo, Sabino De Gisi, Ilenia Farina, Claudio Ferone, Claudia Labianca, Andrea Petrella, Raffaele Cioffi, Michele Notarnicola

**Affiliations:** 1 Department of Civil, Environmental, Land, Building Engineering and Chemistry (DICATECh), Polytechnic University of Bari, Via E. Orabona n. 4, 70125 Bari, Italy; 2 Department of Engineering and INSTM Research Unit, University of Naples “Parthenope”, Centro Direzionale, Isola C4, 80143 Naples, Italy; 3 Department of Civil and Environmental Engineering, The Hong Kong Polytechnic University, Hung Hom, Kowloon, Hong Kong, China

**Keywords:** green remediation, geopolymer, wet/cold granulation, cement-free stabilization/solidification, waste valorization, metal leachability

## Abstract

Stabilization/solidification (S/S) is becoming increasingly important, as it allows the remediation of contaminated sediments and their recovery into materials for civil engineering. This research proposes a cement-free cold granulation process for manufactured low-cost aggregates from marine sediments contaminated with organic compounds and metals. After the chemo-physical characterization of the study materials, two mix designs were prepared in a rotary plate granulator by adding two industrial by-products as geopolymer precursors, coal fly ash (CFA) and Blast Furnace Slag (BFS), but also alkaline activation solutions, water, and a fluidizer. The results indicated that sediments treated with mix 1 (i.e., with a higher percentage of water and fluidifier) represent the optimal solution in terms of metal leachability. The metal leachability was strictly influenced by aggregates’ porosity, density, and microstructure. The technical performance (such as the aggregate impact value > 30%) suggested the use of granules as lightweight aggregates for pavement construction. The results indicated that cold granulation represents a sustainable solution to recycling contaminated marine sediments, CFA, and BFS into lightweight artificial aggregates.

## 1. Introduction

Anthropogenic activities involve the production of several hazardous pollutants which may pose a serious concern to the ecosystem. For example, marine-sediment-bound pollutants can enter aquatic food chains and have adverse effects on ecosystems and human health [1].

Several remediation technologies have been developed to clean up marine sediments contaminated by organic and inorganic pollution, including both ex situ and in situ options [2].

Among the ex-situ remediation techniques, stabilization/solidification (S/S) has captured extensive interest not only for being reliable and cost-efficient [3], but also because it represents a key technology for producing new engineering materials by treatment and reuse of dredged contaminated sediments [4]. S/S involves the addition of binders and additives to a contaminated material in order to immobilize pollutants, but also to improve its mechanical behavior [5,6]. The treated sediment can be recycled in road construction [7], cemented mortars [8], fill material and blocks [4], and raw materials for brick production [9], or at least disposed in sanitary landfills for unhazardous waste. In fact, dredged materials are often classified as waste and disposed of in costly and environmentally risky ways.

Recently, a new environmental sediment S/S approach that utilizes industrial/agricultural by-products received particular attention [10,11]. In a broad view of “green remediation”, this simultaneously addresses the problem of contaminated sediments while supporting material recycling and mitigation of environmental impacts (e.g., carbon footprint and landfill disposal) [12]. Wang et al. [13] investigated the efficacy of wood waste biochar as a green admixture in S/S. The biochar addition allowed the immobilization of toxic elements and organic contaminants present in the sediment, which became suitable for recycling as a fill material and in paving blocks. Shubbar et al. [14] developed a low-carbon binder from the ternary blending of cement, ground granulated blast furnace slag, and high-calcium fly ash. This increased the mechanical strengths of the treated sediment thanks to the high glassy silica and alumina content [11].

However, applying S/S treatments does not allow obtaining a ready-to-use product in civil applications. In this regard, granulation processes represent viable options for obtaining a significant particle size enlargement using a rotary drum or similar device [15]. Recently, only a few studies have focused on coupling the two techniques to obtain aggregates from the treatment of waste materials such as contaminated sediment and soil [16], municipal solid waste and incinerator bottom ashes [17], and automotive shredder residues [18]. According to the final purpose, granulation processes can manufacture artificial aggregates. Ren et al. [19] defined granulation as any process of consolidating solid particles in a size range of 1–500 μm into aggregates of larger sizes, ranging from 2 mm to 10 mm. The result consists of granules whose characteristics depend on the chosen formulation and the design process adopted (i.e., type of granulator, operating parameters, etc.).

Several authors studied the granulation process with traditional binders (e.g., Portland cement) to produce artificial aggregates [20]. This process has economic and environmental advantages by requiring less energy (process carried out at room temperature) than energy-intensive industrial alternatives, such as polymerization [21]. Only recently, binders with reduced CO_2_ content, such as geopolymers and alkali-activated materials, have been proposed for S/S processes [22,23,24]. These alternatives have gained increasing interest from researchers due to promising results in terms of physical, mechanical, and durability properties [25]. Geopolymers represent a class of amorphous aluminosilicate binder materials produced by the reaction of an aluminosilicate source with a concentrated aqueous solution of an alkali metal silicate or hydroxide. Geopolymer materials can provide performances comparable to that obtained with traditional cement, but with a significant reduction in greenhouse gas emissions and good fire and acid resistance [26,27,28].

The literature shows how the environmental applications of the granulation process with geopolymers still need to be explored, despite their high potential, especially in the case of stabilization/solidification of hazardous waste [29,30]. In this context, this study aimed to contribute to the research of sustainable S/S treatments by exploring the feasibility of cement-free granulation for the recycling of organically and inorganically contaminated marine sediments. In particular, to provide scientific insights into waste recycling, this study aimed at transforming contaminated materials into a chemically and physically stable material, suitable for engineering applications, via a cold granulation process based on low-cost geopolymers.

For this reason, compositional and microstructural analyses were conducted to characterize the contaminated materials and treatment reagents: coal fly ash (CFA) and Blast Furnace Slag (BFS). Moreover, the environmental and technical aspects of the recycled aggregates were evaluated by leaching, physical-mechanical tests, and microstructural analysis.

The novelty of the study lies in the application of alkaline reagents and industrial waste (i.e., CFA and BFS) as a S/S technique to producing low-cost aggregates from contaminated marine sediments. Additionally, the experimental investigation was characterized by the absence of pre-treatment of the sampled sediments and by the use of a rotary plate granulator plant that is suitable for scaling up the results.

## 2. Materials and Methods

### 2.1. Framework of the Experimentation

**Figure 1:**
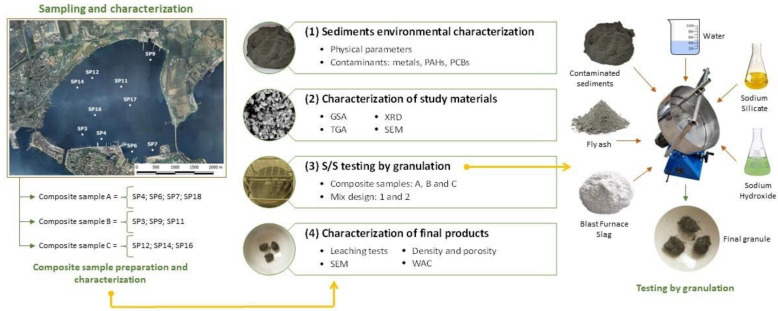
Overview of the experimental framework.

The investigation aimed to obtain environmental-friendly recycled aggregates through stabilization technology by a cold granulation process. The study involved the following four main phases: (i) chemical characterization of ten samples of marine sediments and realization of three composite samples; (ii) characterization of the study’s materials (i.e., fly ash and composite marine sediments); (iii) S/S testing by granulation; (iv) evaluation of the chemical-physical characteristics of the final products (Figure 1). First, an environmental characterization of the sediments was necessary to prepare the composite samples under study. Next, composite marine sediments and geopolymer precursors were characterized by grain size analysis (GSA), thermogravimetric analysis (TGA), X-ray diffraction mineralogical analysis, and scanning electron microscopy (SEM) observations. Finally, after 28 days of maturation, leaching tests and physical characterization analyses were performed on the granulated materials.

The study presented in this article is part of a project promoted by the “Special Commissioner for urgent measures of reclamation, environmental improvements and redevelopment of Taranto” (appointed by the Italian government), which is aimed at the selection of sustainable strategies for the remediation of the Mar Piccolo, which is considered one of the most polluted marine ecosystems in Europe [31].

### 2.2. Contaminated Marine Sediments

Contaminated marine sediments were sampled from the first basin of the Mar Piccolo of Taranto (Southern Italy, Figure 1). A team of expert scuba divers carried out the sampling campaign in the first 50 cm of the seafloor [32]. These sediments are mainly silty clays of very high plasticity and activity, and their liquidity index is on average larger than one [33]. As also discussed by Vitone et al. [34], X-ray diffractometry of them shows the widespread presence of phyllosilicates (such as interstratified illite/smectite, chlorite/smectite, kaolinite, and chlorite); minor constituents are quartz, feldspars, and carbonates (calcite and dolomite, and aragonite is present in trace amounts).

Until characterization, each sample was stored at +4 °C to avoid alteration of the sediment’s physical-chemical properties. For this purpose, the standard protocols of ISPRA (the Italian Institute for the Environmental Protection and Research) were used. The concentrations of metals were obtained by ICP-OES measurements (inductively coupled plasma-optical emission spectrometry) in accordance with the EPA method 200.8. For the determination of the organic contaminants, a gas chromatograph–mass spectrometer (GC-MS) and the EPA method 8275A were used. The representative samples for the subsequent S/S tests were created by mixing homogeneous sediments in terms of levels and types of contamination, as shown in Figure 1.

### 2.3. Geopolymers Precursors and Activator Solutions

Coal fly ash (CFA)—named EFA-Füller HP—from the BauMineral power plant (Heyden, Germany) was used as a geopolymer precursor. It is a fine-grained pozzolanic binder that consists mainly of SiO_2_ and Al_2_O_3_ according to the DIN EN 450 (the content of reactive SiO_2_ is at least 25% by mass). Instead, Blast Furnace Slag (BFS) from Italcementi (Bari, Italy) was used to accelerate the setting process. The BFS is high in iron oxide (25.5%), calcium oxide (17.5%), and alumina (8.9%) [35]. CFA and BFS were employed as residues from industrial plants operating in Italy. Moreover, two activating solutions, prepared with sodium silicate (Na_2_SiO_3_ with initial silica modulus ratio of 2.0) from Prochin Italia Srl (Napoli, Italy) and sodium hydroxide (15 M NaOH) (Sigma-Aldrich, Milano, Italy), were used. These strong molar solutions were chosen to improve the degree of polymerization [36].

### 2.4. Composition and Microstructural Analysis

A particle-size analysis was performed using the laser diffractor Mastersizer 3000 by Malvern Panalytical Ltd. (Malvern, UK) (particle size range: 0.01 µm ÷ 3500 µm) with the Hydro EV wet sample dispersion unit.

A mineralogical characterization of the samples was performed using the X-ray diffraction (XRD) instrument Miniflex 600 (Rigaku Corporation, Tokyo, Japan) equipped with a diffracted beam monochromator set for Cu-Kα radiation (λ = 1.5418 Å). The 2θ scan range was 5 ÷ 80° with a step size of 0.02° and a scanning speed equal to 2θ/s. The mineralogical phase recognition was performed using Rigaku PDXL2 software (Rigaku Corporation, Tokyo, Japan) and the PDF4+ database (International Centre for Diffraction Data, Tokyo, Japan).

TGA was conducted using the TGA/DSC 2 (Mettler Toledo, Columbus, OH, USA), which is a combined device capable of measuring sample mass change and heat flow simultaneously. Each sample was tested within a temperature range of 25 ÷ 1000 °C with a heating rate of 10 °C/min.

The microstructures of the raw materials and final products were studied by means of SEM investigations. The morphological analysis was carried out using a ProX microscope (Phenom, Kondapur, India) equipped with the EDS (Energy Dispersive Spectroscopy) microprobe and the ProSuite software for elemental analysis evaluation.

### 2.5. S/S Testing and Granule Characterization

Composite sediment samples were air-dried till reaching the optimal water content (maximum moisture content 20%). The S/S mixtures were prepared using different types and contents of geopolymer precursors based on industrial wastes (Table 1). The mixture designs were chosen according to the results of the RILEM technical committee TC 247-DTA [37]. In particular, the mixtures were designed to achieve suitable chemo-physical characteristics for reuse via a low water/binder ratio (0.2 ÷ 1.6). Indeed, as the marine sediments from Mar Piccolo were highly contaminated, a strong molar solution of sodium hydroxide (15 M) was used as geopolymer precursors activator [36].

The experimentation involved the use of a rotary plate granulator able to simulate a granulation process for aggregate production. The granulation tests allowed the optimization of the mixtures in terms of: (i) percentage of non-granulated powder (<5%); (ii) granulation starting time (4 ÷ 5 min); (iii) target granule size (4 ÷ 18 mm) according to the European Standard EN 13055’s [38] specifications. In this regard, only the mix design with a geopolymer precursors/sediment ratio of 0.88 was considered adequate and was optimized to investigate the influence of water content (Table 1). The weight ratio CFA:BFS:sediment was 6:1:8 for all the specimens. Moreover, the mixtures differed both in the sodium silicate dose (Na_2_SiO_5_ on 100 g of precursor), which was 26.9 for the former and 31.3 for the latter, and in the sodium hydroxide dose (NaOH on 100 g of precursor)—2.7 and 4.9, respectively.

The curing phase was conducted at room temperature and 100% humidity for 28 days. The treated materials underwent the leaching test at the end of the curing process [39]. A 40 g portion was sampled and transferred to a polyethylene bottle. Demineralized water was added with a solid–liquid ratio of 1:10 by weight, and the bottles were kept in rotation at 10 rpm for 24 h using an overhead mixer. After 24 h, the leachate was filtered to remove suspended solids and chemically characterized according to appropriate standard methods [40]. The heavy metals’ concentrations were determined by using ICP-OES.

The morphology of the granules was studied by means of SEM images, acquired with the method described in Section 2.4. In addition, after the curing time, the granules were analyzed to investigate their physical properties. The determination of apparent density, porosity, and water absorption capacity (WAC) was carried out according to the UNI 11060 [41]. In particular, WAC at atmospheric pressure was determined using the following relation (Equation (1)):

WAC = 100 × (m_sat_ − m_dry_)/m_dry_ (1)

where m_sat_ is the water-saturated mass in air and m_dry_ is the dry mass, after drying in the oven at 60 °C. Tests were conducted in triplicate.

The samples were also tested to determine the aggregate impact value (AIV) to assess their suitability in concrete. The experiment was performed according to the British Standard 812 [42], which gives a relative measure of the resistance of an aggregate to sudden shock or impact. The AIV is a measure of the resistance gradually applied as a compressive load. First, the aggregates were washed and dried at a constant temperature in the range of 105 to 110 °C, and then the samples were cooled down to room temperature. After that, the aggregates were weighed, placed into the cylinder, and then transferred to the cup where they were compacted using the tamping rod. The hammer was released to fall freely on the aggregates. The sediment samples were subjected to 15 blows. They were removed from the cup and sieved through a 2.36 mm sieve. The particles passing through the 2.36 mm sieve were weighed to detect weight loss. The experiment was repeated for all the mixtures prepared.

## 3. Results

### 3.1. Contaminated-Marine-Sediment Characterization

The particle-size characterization of the sediment samples revealed fine-grained soils, in which the fine fraction (<0.063 mm) varied between 35.16% (SP4) and 91.76% (SP9), the sand fraction (2 ÷ 0.063 mm) ranged from 4.28% of the SP9 sample to 44.16% of the SP4 sample, and the gravel fraction (>2 mm) was between 3.96% (SP9) and 31.76% (SP12). Contaminant concentrations (Table 2) were compared with the corresponding limits set by site-specific regulations [43].

Regarding inorganic contamination, copper (Cu), lead (Pb), mercury (Hg), and zinc (Zn) exceeded the corresponding limit values in all the samples analyzed. Arsenic (As) concentrations were higher than the threshold limit values in the samples SP4, SP6, and SP7. Instead, cadmium (Cd) concentration exceeded the regulation limit in the samples SP4 and SP14. SP4, SP6, SP7, and SP17 samples were characterized by concentrations of polycyclic aromatic hydrocarbons (PAHs) higher than the limit values (11,652, 6552, 5810, and 4515 µg/kg, respectively). The cumulative polychlorinated biphenyls (PCBs) concentration exceeded the threshold limits in all samples. The maximum values were found in the SP6 and SP7 (22,253 and 19,283 µg/kg, respectively).

The representative samples for the subsequent S/S tests were prepared by mixing the sediments with similar characteristics in terms of level and type of contamination. The most contaminated sediments were found in the samples SP4, SP6, SP7, and SP17, so they were used to produce the composite sample named “A”. The other sediment samples were used for other two composite samples named “B” (SP3, SP9, and SP11) and “C” (SP12, SP14, and SP16) with lower levels of contamination than “A”. The results of the composite samples’ characterizations are reported in Table 3.

**Figure 2:**
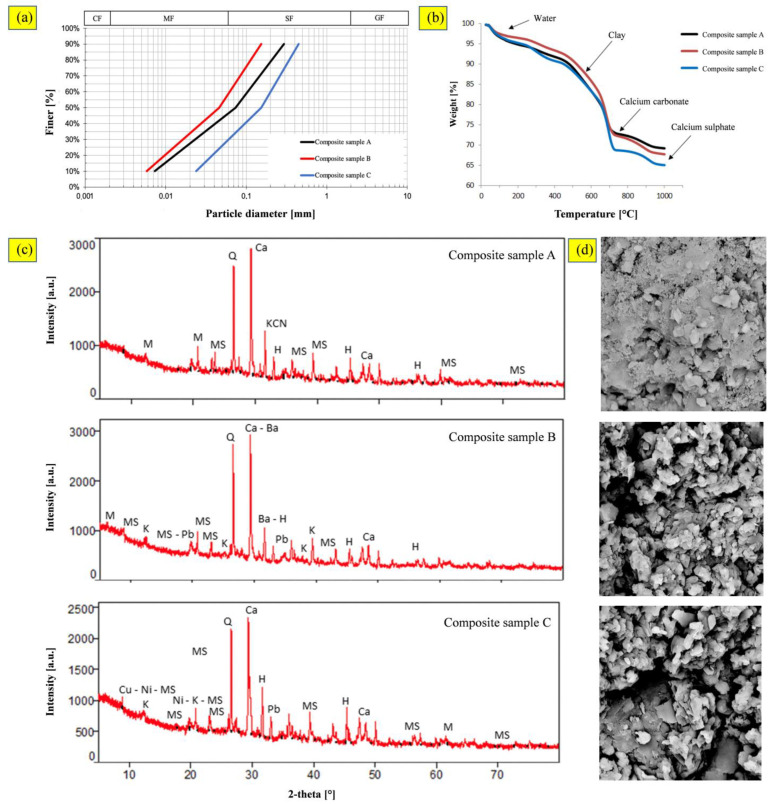
Sediments: (**a**) particle size distribution; (**b**) TGA curves; (**c**) XRD spectra; (**d**) SEM images (scale 10,000:1).

According to the particle size distribution (Figure 2a), the samples were classified as sand with silt (Dv_50_ = 74 µm), silt with sand (Dv_50_ = 46 µm), and silty sand (Dv_50_ = 154 µm) for composite samples A, B, and C, respectively.

It is pointed out that the particle size of the untreated material has a key role in the granulation process. Solids with variable dimensions gave dense granules because smaller particles can fill the pores between larger particles. Furthermore, impeller speed, granulation time, and the amount and viscosity of the liquid binder may each have a significant effect on the granulation process [44].

The TGA showed a similar trend for all the composite samples (Figure 2b). The weight losses (30 ÷ 35%) were mainly due to water (hygroscopic and capillary) and decomposition of the organic matter. Four stages were found in the degradation process of this sediment, mainly occurring at the temperature ranges of 50–200 °C, 200–500 °C, 500–700 °C, and 700–1000 °C. The first stage corresponds to the chemically bound water and natural organic matter [45]. In the second stage, the weight loss might be associated with decomposition of clays and oxidation of organic matter—low-molecular-weight hydrocarbons are released and burned [46]. The third stage is mainly due to the combustion of different and more recalcitrant classes of organic matter. In the last stage, most carbonate-associated inorganic compounds were completely degraded; the loss at about 900 °C was presumably due to the thermal decomposition of calcium sulphate [47]. Sample C followed a slightly different pattern at temperatures around 700–1000 °C, and had greater weight loss than samples A and B.

The presence of clay minerals was confirmed by the XRD analysis (Figure 2c). The diffractograms showed a mixture of main crystalline phases corresponding to the diffraction patterns of quartz, calcine, halite, and clay minerals (i.e., a mixture of montmorillonite, kaolinite-montmorillonite, montmorillonite-chlorite, kaolinite, and muscovite). Sulphates, phosphates, and heavy metals were identified as minority phases. Moreover, hematite was significantly present, dragged mainly by the wind from the iron ores heaps of the near steel factory [48].

Morphological analyses, performed on the sediments after drying, revealed a more homogeneous and compact aspect of sample A (Figure 2d). Instead, the SEM images highlighted a very similar structure for the composite samples B and C, and an irregular morphology of the constituent particles was evident.

### 3.2. Characterization of Geopolymers’ Precursors 

#### 3.2.1. Coal Fly Ash

**Figure 3:**
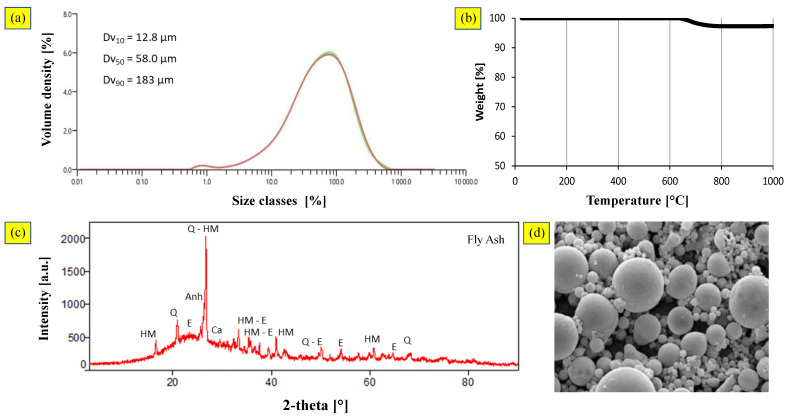
Coal fly ash: (**a**) particle size distribution; (**b**) TGA curves; (**c**) X-ray diffraction pattern; (**d**) SEM image (scale 1000:1).

The particle size of CFA ranged between 12.8 and 183 μm (Figure 3a), implying high combustion efficiency [49]. From the particle distribution, it was possible to determine the sizes of the 10%, 50%, and 90% (by volume) of the smallest particles, denoted Dv_10_, Dv_50_, and Dv_90_, respectively. The span, which is calculated as (Dv_90_ − Dv_10_)/Dv_50_, was 2.93, and showed the usability of the ashes; a uniform particle size distribution would favor a layering mechanism of smaller particles onto the surfaces of larger ones in the granulation phase, leading to an uneven growth process [50].

The thermogravimetric analysis results highlighted that the weight loss started at 650 °C and was completed at 800 °C. The overall weight loss was <5%, which can be attributed to removing the unburnt (Figure 3b). The mineralogical characterization (Figure 3c) showed the presence of quartz (SiO_2_), mullite (A_l6_Si_2_O_13_), and hematite (Fe_2_O_3_). Anhydrite (CaSO_4_) and calcite (CaCO_3_) were detected as minority phases, likely resulting from the abatement treatments of pollutants in the gas. SEM images (Figure 3d) showed the typical spherical morphology of fly ash particles, caused by the high-temperature melting and solidification processes during transport in the flue gas.

#### 3.2.2. Blast Furnace Slag

**Figure 4:**
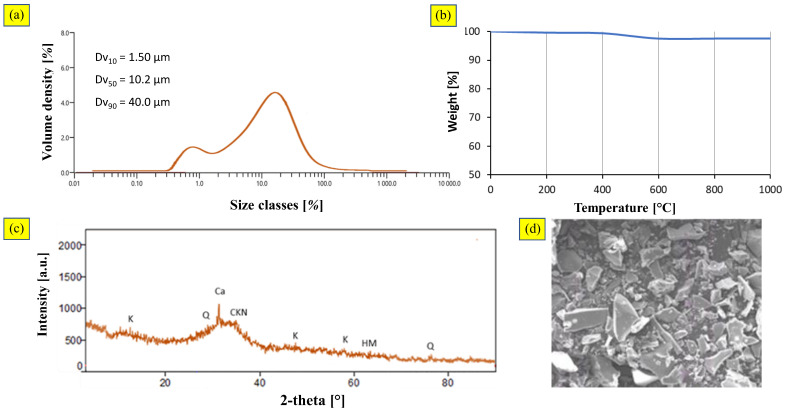
Blast Furnace Slag: (**a**) particle size distribution; (**b**) TGA curves; (**c**) X-ray diffraction pattern; (**d**) SEM image (scale 1000:1).

Figure 4a shows the particle size distribution curve of BFS, which was measured with the laser-diffraction technique. From the particle-size distribution, a mean equivalent spherical particle diameter of 18 µm (Dv_50_) was observed. In addition, 10% fell within the range of 0.3–10 µm (Dv_10_), and 90% of particles were less than 40 µm in diameter (Dv_90_). The fineness of BFS is a very important parameter because increased fineness results in better strength development, but in practice, fineness is limited by economic and performance considerations and factors such as setting times and shrinkage [51]. A span of 2.14 was about the same value determined for CFA, showing the compatibility of raw materials for the granulation process [52].

Gradual weight reduction (<3%) in the temperature range 450–600 °C was observed due to the water still present in the sample (Figure 4b). At higher temperatures (above 500 °C), chemical bonds were broken, and the moisture was released and evaporated from the samples. After moisture evaporation, no further mass losses were noticed.

The diffraction pattern (Figure 4c) showed the dominant presence of calcite (CaO) and quartz (SiO_2_). A calcite peak was detected, related to the presence of limestone as a fluxing agent in the parent reactor. The mineralogical characterization indicated that glassy phases (i.e., amorphous structure) predominated.

Figure 4d illustrates the SEM micrographs for raw BFS. Due to the instant cooling of the liquid slag, the particles were irregular, angular, and glassy in appearance.

### 3.3. S/S Performance and Granule Characterization

**Figure 5:**
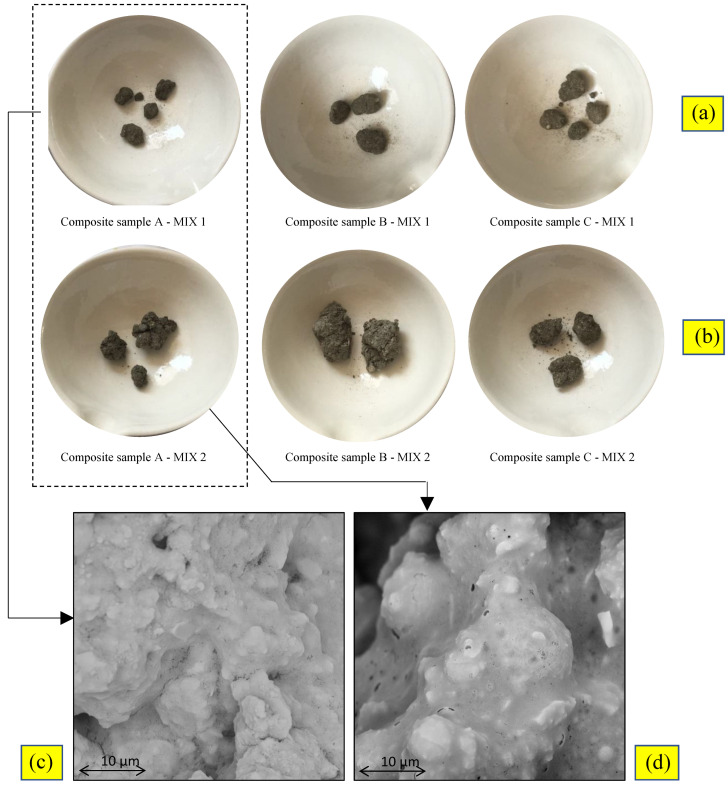
Granules performed by the S/S treatments with mixtures 1 (**a**) and 2 (**b**); SEM images of sample A treated by mixtures 1 (**c**) and 2 (**d**).

Table 4 and Figure 5 show the results of the granulation process: metal concentrations detected in the leached fluid and morphological analysis, respectively.

The first consideration emerging from the analysis of the results was the total absence of organic contaminants in the leachates. However, these contaminants may have a detrimental effect on the properties of treated materials, slowing down the hydration process of binders [29]. Indeed, the leaching tests on the treated composite sample A showed As and Cu release values higher than the limits imposed by the Italian Decree on waste recycling [53]. This implied potential disposal in non-hazardous waste landfills.

Concerning As (Table 4), the release pattern varied with the sediment type: for sample A, the concentrations exceeded the limit values (50 mg/L) for both mix designs; instead, B and C samples showed the lowest concentrations. Similar behavior was found for Cu concentrations. The concentrations of Hg and Pb always resulted in being lower than the instrumental limit of detection (LOD), equal to 0.005 and 0.02 mg/L, respectively. The pattern of Zn release varied both with the sediment sample and mix design: the leachate concentration varied between 75 µg/L (mix 2, composite sediment sample C) and 20 µg/L (mix 1, composite sediment samples B). However, Zn concentrations were below the limit value (3 mg/L).

According to the limits indicated in EN 13055 standard [38], all treated sediments can be classified as lightweight aggregates. Particularly, dry density values appeared lower than 2000 kg/m^3^ (Table 4). It is possible to observe that, going from A to C samples, an increase in density occurred. These changes brought a reduction in both porosity and water absorption capacity. The porosity varied between 3.32% (1_C) and 11.86 (2_A); instead, the WAC ranged from 1.64% of the 1_C sample to 6.56% of the 2_A sample. These parameters seemed to decrease as the activator solutions/geopolymer precursors ratio decreased.

The results of the aggregate impact value (AIV) test are reported in Table 4. Composite samples A, B, and C of mixture 1 exhibited values of 31.43%, 34.21%, and 33.56%, respectively; whereas the values measured for the threes samples of mixture 2 were 32.75%, 34.46%, and 31.83%, respectively. The aggregate impact value, when tested according to the requirements of BS 812-2, shall not exceed 45% for structural concrete; instead, aggregates for concrete wearing surfaces should have an aggregate impact value not greater than 30%. For this reason, it should be noted that the examined sediment aggregates are not suitable as concrete aggregates, but they should be employed for pavement construction.

In agreement with previous works [54], the size of the aggregates increased with water content. However, for all mixtures, the size of the aggregates ranged between 4 and 18 mm, according to EN 13055 [38].

The obtained granules were observed in the SEM in order to define their morphological characteristics. SEM images revealed that the geopolymers binders created a matrix composed mainly of amorphous phases that embedded sediment particles (Figure 5c,d).

Sample A_2 presented unreacted fly ash particles in the mix [55]. This observation demonstrates an incomplete hydration development during the granulation process, which can be associated with lower mechanical results (i.e., AIV). Even if the FA:BFS ratio was kept constant in all the mixtures, activator solutions’ different reactivities influenced the final geo-composites’ microstructure. Further, the addition of a fluidifier to improve the workability formed a more homogeneous microstructure.

The physical characterization confirmed the influence of the microstructure of the aggregates. Regarding mix design 1, the treatment of composite sample A produced granules characterized by a lower density (1800 kg/m^3^), higher porosity (9.17%), and higher WAC (5.05%). For the other two composite samples, the porosity ranged from 4.27% (1_B) to 3.32% (1_C), and similarly, the water absorption ranged from 1.64% (1_C) to 2.27% (1_B) (Table 4). A similar trend was found for mixture 2. This behavior was presumably due to the presence of organic contaminants that inhibited the stabilization/solidification process [56].

## 4. Discussion

The leaching tests indicated that the concentrations of As in the aggregates from composite sample A were of significant concern (respectively, 158 ± 6 mg/L and 175 ± 18 mg/L for mixtures A_1 and A_2). These values exceeded the acceptance criteria for recycling (>50 mg/L). Instead, the metal leachability of all aggregates from composite samples B and C complied with the acceptance criteria.

**Figure 6:**
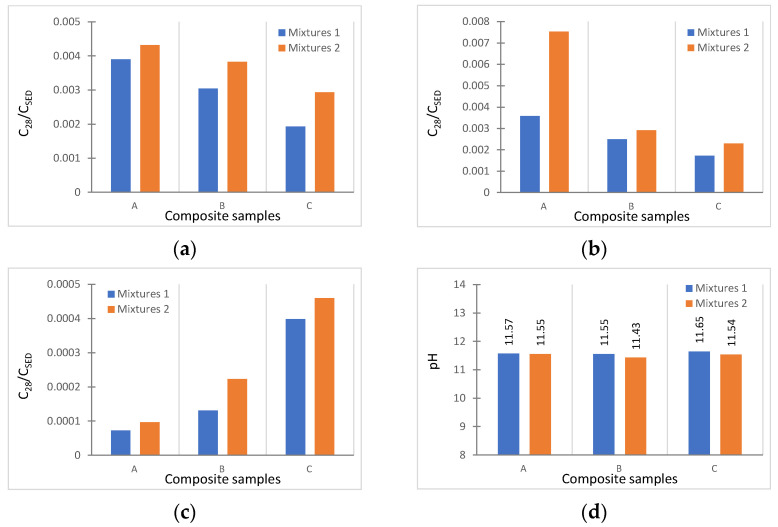
Rates of leaching of metals (C_28_/C_SED_) for As (**a**), Cu (**b**), and Zn (**c**); and pH trend (**d**).

It was possible to calculate the rate of leaching for metals as a ratio of the content of an element in eluate after 28 days (C_28_ in mg/L) to its total content in the untreated sediment (C_SED_ in mg/kg) (Figure 6). This coefficient provides information about both the amount and rate of leaching of a given element from the treated sediment [32]. The data revealed the highest amounts of As, Cu, and Zn were released from sediments treated with mix design 2. It could be noted that with the increase in the content of activator solutions (from 10.8% of mix 1 to 13.3% of mix 2) and the reduction in mixing water (from 11.5% of mix 1 to 9.1% of mix 2, including the fluidifiers), the leaching behavior of the mixture worsened. This confirms what the literature reported [57]; in fact, the increase in sodium hydroxide in the activating solution and the decrease in mixing water produced more porous granules, and therefore, it tended to release more contaminants.

According to the literature [31,58], the pH is one of the main factors influencing the mobility of metals. In this case, the percentage of geopolymer precursors used in the mixtures led to more alkaline values of pH, varying between 11.65 (C_1) and 11.43 (B_2); this appeared significantly dependent on the CaO and MgO mineral contents present in the FA and BFS [59]. Immobilization of heavy metals in a high-pH condition has been reported elsewhere [60,61]. In addition, As were found to be more mobile in the basic environment [62,63].

On the other hand, in this case, the leaching rate of the samples was not only affected by the difference in pH (Figure 6d). The porosity of aggregates, which is dependent on the spaces between coarser fractions, can favor chemical reduction, and therefore, element leaching [54]. In line with this, with regard to As and Cu, Figure 6 shows higher leachability rates for the most porous samples: A (9.17 to 11.86, respectively, for mixes 1 and 2) and B (4.27 to 7.06, respectively, for mixes 1 and 2). In addition, mix 2 (samples A, B, C) showed more metal release overall than mix 1, in line with the corresponding porosity values (Table 4). Consequently, the high mobility of metals from mixture 2 can be ascribed to the physical characteristics of the granules (i.e., density and porosity). As said above, the granules obtained with mixture 2 were characterized by greater porosity.

To reduce the metal leaching from geopolymers, it is also possible to include additives limiting the capillary porosity of the structure, such as titanium nanoxide [64]. Additionally, several studies confirmed improvements in geopolymer properties by hydrothermal treatment [65,66]. Further physical tests (e.g., specific gravity and crushing strength test) could be conducted on the lightweight artificial aggregates in order to assess additional applications in the construction sector (such as coarse aggregate in concrete mixtures) [67,68].

## 5. Conclusions

This research explored the feasibility of recycling contaminated marine sediment and various industrial wastes as resources for producing new materials for civil engineering via stabilization/solidification. Out of several mix designs considered, only the mixtures with a weight ratio for CFA:BFS:sediment = 6:1:8 were deemed adequate and tested. While the granules from composite sediment samples B and C had leaching test values compatible with the production of lightweight artificial aggregates, treated sample A (i.e., with the most contaminated sediment) was destined for landfill disposal for non-hazardous waste. While the granules from composite sediment samples B and C had leaching test values compatible with the production of lightweight artificial aggregates, the granules from sample A (i.e., with the most contaminated sediment) were destined for landfill disposal for non-hazardous waste. The results showed that the metals’ leachability was strictly influenced by sample porosity. Regarding the physical characteristics of granules, the observed values of density, WAC, and AIV were such that the granules can be classified as lightweight aggregates for pavement construction. It can be concluded that the water content influenced the micro-structure of the geopolymer: the smaller the water/binder ratio is, the more unreacted components there are, the more porous the structure is, and the higher the leachability is.

Cement-free S/S by granulation of alkaline reagents demonstrated to be a reliable technique for recycling industrial waste. Future experiments should aim at producing and characterizing low-cost aggregates starting from the results obtained from this investigation.

## Figures and Tables

**Table 1:** Mixture design for S/S treatment (values in dry weight %).

Mix	Activator Solutions			Geopolymer Precursors		Water	Fluidifier	Sediment
	Na_2_SiO_5_	NaOH	H_2_O	CFA	BFS			
1	9.8	1.0	2.0	31.2	5.2	7.2	2.0	41.6
2	11.4	1.8	2.6	31.2	5.2	5.2	1.0	41.6

Note: Na_2_SiO_3_ = sodium silicate; NaOH = sodium hydroxide; H_2_O = water component in activator solutions; CFA = coal fly ash; BFS = Blast Furnace Slag.

**Table 2:** Chemo-physical and particle-size characterizations of natural sediment samples.

	Unit	Natural Sample										Limit Value
		SP3	SP4	SP6	SP7	SP9	SP11	SP12	SP14	SP16	SP17	
Chemo-Physical parameters												
pH	u. pH	9.1	8.9	9.1	9.2	9.2	9.2	8.9	8.8	9.1	9.3	-
Eh	mV	−109.5	−100.9	−106.5	−115.8	−114.6	−117.8	−98.2	−100.9	−116.9	−122.7	-
MC	%	50.83	52.0	50.6	20.2	51.8	53.8	58.4	65.1	51.8	54.2	-
OM	%	19.90	23.4	19.4	20.2	17.1	18.8	12.9	23.7	18.5	17.5	-
Ash	%	29.27	24.6	30.0	59.6	31.1	27.4	28.7	11.2	29.7	28.3	-
GF	%	4.80	20.68	12.87	13.69	3.96	7.41	31.76	18.43	14.39	7.47	-
SF	%	12.20	44.16	14.87	27.72	4.28	15.61	21.97	33.37	12.23	14.76	-
PF	%	83.00	35.16	72.26	58.59	91.76	76.98	46.27	48.20	73.38	77.76	-
Inorganic contaminants												
As	mg/kg	11.6	**31.7**	**48.4**	**65.2**	9.2	13.3	13.4	19.0	11.8	18.3	20.0
Be	mg/kg	0.7	0.8	1.2	1.0	1.0	1.1	0.6	0.9	0.7	1.5	-
Cd	mg/kg	0.4	**2.5**	0.9	1.0	0.5	0.5	0.5	**1.3**	0.4	0.6	1.00
Cr	mg/kg	54.5	81.8	80.8	78.0	75.2	71.6	43.3	65.3	57.1	76.7	160.0
Co	mg/kg	6.5	8.4	9.9	11.1	8.5	7.00	4.9	7.1	5.5	7.9	-
Cu	mg/kg	**53.5**	**172.1**	**121.2**	**131.9**	**58.4**	**64.6**	**62.9**	**117.8**	**55.3**	**80.2**	45.0
Pb	mg/kg	**102.5**	**314.6**	**241.3**	**392.0**	**50.2**	**94.0**	**57.9**	**106.7**	**74.8**	**126.7**	50.0
Hg	mg/kg	**4.2**	**8.1**	**14.3**	**24.0**	**1.2**	**3.5**	**1.5**	**2.5**	**2.6**	**5.9**	0.80
Ni	mg/kg	31.9	40.6	50.1	57.2	48.9	37.5	26.8	44.2	30.4	41.8	100.0
V	mg/kg	56.7	64.0	105.8	75.0	73.6	77.1	62.0	103.2	59.9	84.4	-
Zn	mg/kg	**146.3**	**541.5**	**430.8**	**464.2**	**116.2**	**193.8**	**206.9**	**423.8**	**159.0**	**222.7**	110.0
Organic contaminants												
ΣPAH	µg/kg	2159	**11,652**	**6552**	**5810**	1263	922	714	3172	1902	**4515**	4000
ΣPCB	µg/kg	**277**	**3163**	**22,253**	**19,283**	**2331**	**794**	**607**	**2345**	**1660**	**3184**	190

Note: measurements exceeding the limit value are in bold.

**Table 3:** Chemo-physical and particle-size characterizations of composite samples.

	Unit	Composite Sample (Average ± St. Dev)			Limit Value
		A	B	C	
Chemo-Physical parameters					
pH	pH unit	9.1 ± 0.5	9.1 ± 0.3	9.0 ± 0.4	-
Eh	mV	−111.5 ± 2.9	−114.0 ± 2.2	−105.3 ± 2.6	-
MC	%	44.2 ± 0.5	29.4 ± 0.8	58.4 ± 0.9	-
OM	%	20.3 ± 0.5	18.4 ± 0.8	18.2 ± 0.9	-
Ash	%	35.5 ± 0.5	42.2 ± 0.8	23.4 ± 0.9	-
GF	%	13.7 ± 1.5	5.4 ± 1.5	21.5 ± 1.5	-
SF	%	25.4 ± 1.5	10.7 ± 1.5	22.5 ± 1.5	-
PF	%	60.9 ± 1.5	83.9 ± 1.5	56.0 ± 1.5	-
Inorganic contaminants					
As	mg/kg	**40.5 ± 0.5**	11.5 ± 0.4	15.0 ± 0.3	20.0
Be	mg/kg	1.0 ± 0.1	0.9 ± 0.1	0.7 ± 0.1	-
Ca	mg/kg	**1.2 ± 0.1**	0.5 ± 0.1	0.7 ± 0.1	1.00
Cr	mg/kg	79.2 ± 2.4	65.1 ± 1.6	54.3 ± 0.9	160.0
Co	mg/kg	9.3 ± 0.5	7.3 ± 0.1	5.8 ± 0.4	-
Cu	mg/kg	**127.4 ± 4.6**	**58.9 ± 1.6**	**78.7 ± 2.9**	45.0
Pb	mg/kg	**268.7 ± 1.8**	**82.4 ± 1.1**	**79.8 ± 1.4**	50.0
Hg	mg/kg	**13.0 ± 0.2**	**3.0 ± 0.5**	**2.2 ± 0.6**	0.80
Ni	mg/kg	47.4 ± 1.3	39.4 ± 1.7	33.8 ± 1.8	100.0
V	mg/kg	82.3 ± 1.8	69.1 ± 1.9	75.1 ± 1.5	-
Zn	mg/kg	**664.8 ± 1.6**	**152.1 ± 1.1**	**263.2 ± 1.8**	110.0
Organic contaminants					
ΣPAHs	µg/kg	**7130 ± 70**	1450 ± 65	1930 ± 83	4000
ΣPCBs	µg/kg	**11,950 ± 55**	**1135 ± 40**	**1535 ± 45**	190

Note: measurements exceeding the limit value are in bold.

**Table 4:** Twenty-eight-day performances of aggregates.

Leaching Characteristics of Granules							
Mix	Sample	Parameter	As (µg/L)	Cu (µg/L)	Hg (µg/L)	Pb (µg/L)	Zn (µg/L)
1	A	Leachate concentration	158 ± 6	554 ± 26	<LOD	<LOD	30 ± 1
		Environmental goals ^(a)^	**✗**	**✗**	**✓**	**✓**	**✓**
	B	Leachate concentration	35 ± 14	147 ± 19	< LOD	<LOD	20 ± 1
		Environmental goals ^(a)^	**✓**	**✓**	**✓**	**✓**	**✓**
	C	Leachate concentration	29 ± 1	136 ± 10	<LOD	<LOD	65 ± 1
		Environmental goals ^(a)^	**✓**	**✓**	**✓**	**✓**	**✓**
2	A	Leachate concentration	175 ± 18	957 ± 29	<LOD	<LOD	40 ± 1
		Environmental goals ^(a)^	**✗**	**✗**	**✓**	**✓**	**✓**
	B	Leachate concentration	44 ± 2	172 ± 20	<LOD	<LOD	34 ± 1
		Environmental goals ^(a)^	**✓**	**✓**	**✓**	**✓**	**✓**
	C	Leachate concentration	44 ± 2	181 ± 10	<LOD	<LOD	75 ± 1
		Environmental goals ^(a)^	**✓**	**✓**	**✓**	**✓**	**✓**
**Physical Characteristics of Granules**							
**Mix**	**Sample**	**Parameter**	**Density** **(kg/m^3^)**	**Porosity** **(%)**		**WAC** **(%)**	**AIV** **(%)**
1	A	Test results	1800 ± 53	9.17 ± 0.30		5.05 ± 0.05	32.75 ± 0.12
		Technical goals ^(b)^	**✓**	**✓**		**✓**	**✓**
	B	Test results	1830 ± 55	4.27 ± 0.15		2.27 ± 0.02	34.46 ± 0.13
		Technical goals ^(b)^	**✓**	**✓**		**✓**	**✓**
	C	Test results	1840 ± 55	3.32 ± 0.10		1.64 ± 0.01	34.83 ± 0.12
		Technical goals ^(b)^	**✓**	**✓**		**✓**	**✓**
2	A	Test results	1810 ± 53	11.86 ± 0.35		6.56 ± 0.05	31.43 ± 0.12
		Technical goals ^(b)^	**✓**	**✓**		**✓**	**✓**
	B	Test results	1837 ± 55	7.06 ± 0.20		3.85 ± 0.03	33.21 ± 0.13
		Technical goals ^(b)^	**✓**	**✓**		**✓**	**✓**
	C	Test results	1852 ± 56	4.44 ± 0.15		2.40 ± 0.02	33.56 ± 0.12
		Technical goals ^(b)^	**✓**	**✓**		**✓**	**✓**

Note: (a) Environmental goals: positive (✓) and negative (✗) if metal concentrations are, respectively, lower and higher than law limits [45]; LODs are about 0.0005 and 0.02 mg/L for Hg and Pb, respectively. (b) Technical goals: positive (✓) and negative (✗) if test results are, respectively, lower and higher than the European Standard for lightweight aggregates [31].

## Data Availability

The data presented in this study are available on request from the corresponding author.

## References

[1] Todaro F., Barjoveanu G., De Gisi S., Teodosiu C., Notarnicola M. (2021). Sustainability assessment of reactive capping alternatives for the remediation of contaminated marine sediments. J. Clean. Prod..

[2] Abdel-shafy H.I., Mansour M.S.M. (2016). A review on polycyclic aromatic hydrocarbons: Source, environmental impact, effect on human health and remediation. Egypt. J. Pet..

[3] Kujlu R., Moslemzadeh M., Rahimi S., Aghayani E., Ghanbari F., Mahdavianpour M. (2020). Selecting the best stabilization/solidification method for the treatment of oil-contaminated soils using simple and applied best-worst multi-criteria decision-making method. Environ. Pollut..

[4] Wang L., Kwok J.S.H., Tsang D.C.W., Poon C. (2015). Mixture design and treatment methods for recycling contaminated sediment. J. Hazard. Mater..

[5] Coppola L., Bellezze T., Belli A., Bignozzi M.C., Bolzoni F., Brenna A., Yang F. (2018). Binders alternative to Portland cement and waste management for sustainable construction Part 2. J. Appl. Biomater. Funct. Mater..

[6] Petrella A., Spasiano D., Rizzi V., Cosma P., Race M., De Vietro N. (2018). Lead ion sorption by perlite and reuse of the exhausted material in the construction field. Appl. Sci..

[7] Wang D., Abriak N.E., Zentar R. (2013). Strength and deformation properties of Dunkirk marine sediments solidified with cement, lime and fly ash. Eng. Geol..

[8] Couvidat J., Benzaazoua M., Chatain V., Bouamrane A., Bouzahzah H. (2016). Feasibility of the reuse of total and processed contaminated marine sediments as fine aggregates in cemented mortars. Construct. Build. Mater..

[9] Messina F., Ferone C., Molino A., Roviello G., Colangelo F., Molino B., Cioffi R. (2017). Synergistic recycling of calcined clayey sediments and water potabilization sludge as geopolymer precursors: Upscaling from binders to precast paving cement-free bricks. Constr. Build. Mater..

[10] Rizzi V., D’Agostino F., Gubitosa J., Fini P., Petrella A., Agostiano A., Semeraro P., Cosma P. (2017). An alternative use of olive pomace as a wide-ranging bioremediation strategy to adsorb and recover disperse orange and disperse red industrial dyes from wastewater. Separations.

[11] Tsang D.C., Olds W.E., Weber P.A., Yip A.C. (2013). Soil stabilization using AMD sludge, compost and lignite: TCLP leachability and continuous acid leaching. Chemosphere.

[12] De Gisi S., Todaro F., Mesto E., Schingaro E., Notarnicola M. (2020). Recycling contaminated marine sediments as filling materials by pilot scale stabilization/solidification with lime, organoclay and activated carbon. J. Clean. Prod..

[13] Wang L., Chen L., Tsang D.C., Kua H.W., Yang J., Ok Y.S., Ding S., Hou D., Poon C.S. (2019). The roles of biochar as green admixture for sediment-based construction products. Cement. Concrete. Comp..

[14] Shubbar A.A., Jafer H., Dulaimi A., Hashim K., Atherton W., Sadique M. (2018). The development of a low carbon binder produced from the ternary blending of cement, ground granulated blast furnace slag and high calcium fly ash: An experimental and statistical approach. Constr. Build. Mater..

[15] Capobianco O., Costa G., Baciocchi R. (2014). Assessment of the operating windows of a combined solidification/stabilization and granulation treatment applied to industrial soil in the context of brownfield regeneration. WIT Trans. Eco. Environ..

[16] Nakhaei A., Marandi S.M., Kermani S.S., Bagheripour M.H. (2012). Dynamic properties of granular soils mixed with granulated rubber. Soil Dyn. Earthq..

[17] Cioffi R., Colangelo F., Montagnaro F., Santoro L. (2011). Manufacture of artificial aggregate using MSWI bottom ash. J. Waste Manag..

[18] Di Palma L., Medici F., Vilardi G. (2015). Artificial aggregate from non metallic automotive shredder residue. Chem. Eng. Trans..

[19] Ren P., Ling T.C., Mo K.H. (2020). Recent Advances in Artificial Aggregate Production. J. Clean. Prod..

[20] Vasugi V., Ramamurthy K. (2014). Identification of design parameters influencing manufacture and properties of cold-bonded pond ash aggregate. Mater. Design.

[21] Kang X., Bate B., Chen R.P., Yang W., Wang F. (2019). Physicochemical and mechanical properties of polymer-amended kaolinite and fly ash–kaolinite mixtures. J. Mater. Civ. Eng..

[22] Lirer S., Liguori B., Capasso I., Flora A., Caputo D. (2017). Mechanical and chemical properties of composite materials made of dredged sediments in a fly-ash based geopolymer. J. Environ. Manage.

[23] Reddy V.A., Solanki C.H., Kumar S., Reddy K.R., Du Y.J. (2019). New ternary blend limestone calcined clay cement for solidification/stabilization of zinc contaminated soil. Chemosphere.

[24] Roviello G., Menna C., Tarallo O., Ricciotti L., Ferone C., Colangelo F., Cioffi R. (2015). Preparation, structure and properties of hybrid materials based on geopolymers and polysiloxanes. Mater. Design.

[25] Molino B., De Vincenzo A., Ferone C., Messina F., Colangelo F., Cioffi R. (2014). Recycling of clay sediments for geopolymer binder production. A new perspective for reservoir management in the framework of Italian legislation: The Occhito reservoir case study. Materials.

[26] El-Eswed B.I., Yousef R.I., Alshaaer M., Hamadneh I., Al-Gharabli S.I., Khalili F. (2015). Stabilization/solidification of heavy metals in kaolin/zeolite based geopolymers. Int. J. Miner. Process..

[27] Jin M., Zheng Z., Sun Y., Chen L., Jin Z. (2016). Resistance of metakaolin-MSWI fly ash based geopolymer to acid and alkaline environments. J. Non-Cryst. Solids.

[28] Colangelo F., Farina I., Travaglioni M., Salzano C., Cioffi R., Petrillo A. (2021). Innovative Materials in Italy for Eco-Friendly and Sustainable Buildings. Materials.

[29] Almadani M., Razak R.A., Abdullah M.M.A.B., Mohamed R. (2022). Geopolymer-Based Artificial Aggregates: A Review on Methods of Producing, Properties, and Improving Techniques. Materials.

[30] Takaluoma E., Samarina T. (2022). Granulation techniques of geopolymers and alkali-activated materials. Alkali-Activated Materials in Environmental Technology Applications.

[31] Todaro F., De Gisi S., Notarnicola M. (2018). Sustainable remediation technologies for contaminated marine sediments: Preliminary results of an experimental investigation. Environ. Eng. Manag. J..

[32] Todaro F., De Gisi S., Notarnicola M. (2020). Contaminated marine sediment stabilization/solidification treatment with cement/lime: Leaching behaviour investigation. Environ. Sci. Pollut. Res. Int..

[33] Cotecchia F., Vitone C., Sollecito F., Mali M., Miccoli D., Petti R., Milella D., Ruggieri G., Bottiglieri O., Corbelli V. (2021). A geo-chemo-mechanical study of a highly polluted marine system (Taranto, Italy) for the enhancement of the conceptual site model. Sci. Rep..

[34] Vitone C., Federico A., Puzrin A.M., Ploetze M., Carrassi E., Todaro F. (2016). On the geotechnical characterisation of the polluted submarine sediments from Taranto. Environ. Sci. Pollut. Res..

[35] Petrillo A., Colangelo F., Farina I., Travaglioni M., Salzano C., Cioffi R. (2022). Multi-criteria analysis for Life Cycle Assessment and Life Cycle Costing of lightweight artificial aggregates from industrial waste by double-step cold bonding palletization. J. Clean. Prod..

[36] Perumal P., Hasnain A., Luukkonen T., Kinnunen P., Illikainen M. (2021). Role of Surfactants on the Synthesis of Impure Kaolin-Based Alkali-Activated, Low-Temperature Porous Ceramics. Open Ceram..

[37] Provis J.L., Arbi K., Bernal S.A., Bondar D., Buchwald A., Castel A., Chithiraputhiran S., Cyr M., Dehghan A., Dombrowski-Daube K. (2019). RILEM TC 247-DTA round robin test: Mix design and reproducibility of compressive strength of alkali-activated concretes. Mater. Struct..

[38] (2016). Lightweight Aggregates.

[39] (2002). Characterisation of Waste—Leaching—Compliance Test for Leaching of Granular Waste Materials and Sludges.

[40] EPA (1994). EPA Method 200.8: Determination of Trace Elements in Waters and Wastes by Inductively Coupled Plasma—Mass Spectrometry.

[41] (2003). Cultural Heritage—Natural and Artificial Stones.

[42] (1960). Methods for Sampling and Testing of Mineral Aggregates Sands and Filters.

[43] ICRAM (2004). Values of Intervention for Sediments of Areas Strongly Anthropized with Reference to the Site of Reclamation of National Interest in Taranto.

[44] Abora K., Beleña I., Bernal S.A., Dunster A., Nixon P.A., Provis J.L., Tagnit-Hamou A., Winnefeld F. (2014). Durability and testing–Chemical matrix degradation processes. Alkali Activated Materials.

[45] Gaál F., Szöllősy I., Arnold M., Paulik F. (1994). Determination of the organic matter, metal carbonate and mobile water in soils simultaneous TG, DTG, DTA and EGA techniques. J. Therm. Anal. Calorim..

[46] Oudghiri F., García-Morales J.L., Rodríguez-Barroso M.R. (2015). Evaluation of Sediments Decontamination by Chelating Agents using Thermogravimetric Analysis. Int. J. Environ. Res..

[47] Bensharada M., Telford R., Stern B., Gaffney V. (2021). Loss on ignition vs. thermogravimetric analysis: A comparative study to determine organic matter and carbonate content in sediments. J. Paleolimnol..

[48] Labianca C., De Gisi S., Todaro F., Notarnicola M. (2020). DPSIR model applied to the remediation of contaminated sites. A case study: Mar Piccolo of Taranto. Appl. Sci..

[49] Sarkar A., Rano R., Mishra K.K., Sinha I.N. (2005). Particle size distribution profile of some Indian fly ash—A comparative study to assess their possible uses. Fuel Process. Technol..

[50] Wang Z., Cao J., Li W., Wang Y., Luo G., Qiao Y., Zhang Y., Xu B. (2021). Using a material database and data fusion method to accelerate the process model development of high shear wet granulation. Sci. Rep..

[51] Siddique R., Cachim P. (2018). Waste and Supplementary Cementitious Materials in Concrete.

[52] FAO (1988). Italian Ministerial Decree Identification of not-hazardous waste subjected to simplified recovery procedures. Ital. Off. J..

[53] Colangelo F., Messina F., Di Palma L., Cioffi R. (2017). Recycling of non-metallic automotive shredder residues and coal fly-ash in cold-bonded aggregates for sustainable concrete. Compos. B Eng..

[54] Hattaf R., Benchikhi M., Azzouzi A., El Ouatib R., Gomina M., Samdi A., Moussa R. (2021). Preparation of Cement Clinker from Geopolymer-Based Wastes. Materials.

[55] Pan Y., Rossabi J., Pan C., Xie X. (2019). Stabilization/solidification characteristics of organic clay contaminated by lead when using cement. J. Hazard. Mater..

[56] Shi C., Fernández-Jiménez A. (2006). Stabilization/solidification of hazardous and radioactive wastes with alkali-activated cements. J. Hazard. Mater..

[57] Kogbara R.B., Al-Tabbaa A., Yi Y., Stegemann J.A. (2012). pH-dependent leaching behaviour and other performance properties of cement-treated mixed contaminated soil. J. Environ. Sci..

[58] Komonweeraket K., Cetin B., Aydilek A.H., Benson C.H., Edil T.B. (2015). Effects of pH on the leaching mechanisms of elements from fly ash mixed soils. Fuel.

[59] De Gisi S., Romaniello L., Dalessandro M., Todaro F., Notarnicola M. (2019). Recovery of iron rich residues from integrated steel making process by hydrated lime/molasses pressurised cold agglomeration. J. Clean. Prod..

[60] Arfania H., Asadzadeh F. (2015). Mobility of heavy metals after spiking in relation to sediment and metal properties: Leaching column study. J. Soils Sediment.

[61] Tigue A.A.S., Malenab R.A.J., Dungca J.R., Yu D.E.C., Promentilla M.A.B. (2018). Chemical Stability and Leaching Behavior of One-Part Geopolymer from Soil and Coal Fly Ash Mixtures. Minerals.

[62] Zhang J., Provis J.L., Feng D., van Deventer J.S.J. (2008). Geopolymers for immobilization of Cr6+, Cd2+, and Pb2+. J. Hazard. Mater..

[63] Xu H., Gong W., Syltebo L., Izzo K., Lutze W., Pegg I.L. (2014). Effect of blast furnace slag grades on fly ash based geopolymer waste forms. Fuel.

[64] Łach M., Korniejenko K., Walter J., Stefańska A., Mikuła J. (2020). Decreasing of Leaching and Improvement of Geopolymer Properties by Addition of Aluminum Calcium Cements and Titanium Oxide. Materials.

[65] Łach M., Hebdowska-Krupa M., Komar N. (2019). Strength and leachability of geopolymers with the addition of municipal solid waste ashes. IOP Conf. Ser. Mater. Sci. Eng..

[66] Li Y.-Y., Zhang T.-T., Jia S.B., Liu J., Quan X.-H., Zheng W. (2019). Mechanical properties and leaching characteristics of geopolymer-solidified/stabilized lead-contaminated soil. Adv. Civ. Eng..

[67] Gesoğlu M., Güneyisi E., Alzeebaree R., Mermerdaş K. (2013). Effect of silica fume and steel fiber on the mechanical properties of the concretes produced with cold bonded fly ash aggregates. Constr. Build. Mater..

[68] Güneyisi E., Gesoğlu M., Mohamadameen A., Alzeebaree R., Algın Z., Mermerdaş K. (2018). Enhancement of shrinkage behavior of lightweight aggregate concretes by shrinkage reducing admixture and fiber reinforcement. Constr. Build. Mater..

